# Design and Testing of an Agricultural Implement for Underground Application of Rodenticide Bait

**DOI:** 10.3390/s150102006

**Published:** 2015-01-16

**Authors:** Hugo Malón, A. Javier Aguirre, Antonio Boné, Mariano Vidal, F. Javier García-Ramos

**Affiliations:** Superior Polytechnic School, University of Zaragoza, Huesca 22071, Spain; E-Mails: hml@unizar.es (H.M.); javier.aguirre@unizar.es (A.J.A.); anbone@unizar.es (A.B.); vidalcor@unizar.es (M.V.)

**Keywords:** FEM, strain gauge, pocket gopher, plough

## Abstract

An agricultural implement for underground application of rodenticide bait to control the Mediterranean pocket gopher (*Microtus Duodecimcostatus*) in fruit orchards has been designed and tested. The main objective of this research was to design and test the implement by using the finite element method (FEM) and considering a range of loads generated on most commonly used furrow openers in agricultural implements. As a second step, the prototype was tested in the field by analysing the effects of forward speed and application depth on the mechanical behaviour of the implement structure. The FEM was used in the design phase and a prototype was manufactured. The structural strains on the prototype chassis under working conditions were tested by using strain gauges to validate the design phase. Three forward speeds (4.5, 5.5, and 7.0 km/h), three application depths (0.12, 0.15, and 0.17 m), and two types of soil (clayey-silty-loam and clayey-silty-sandy) were considered. The prototype was validated successfully by analysing the information obtained from the strain gauges. The Von Mises stresses indicated a safety coefficient of 1.9 for the most critical load case. Although both forward speed and application depth had a significant effect on the stresses generated on the chassis, the latter parameter critically affected the structural behaviour of the implement. The effects of the application depth on the strains were linear such that strains increased with depth. In contrast, strains remained roughly constant regardless of variation in the forward speed.

## 1. Introduction

Different types of moles and pocket gophers can cause damage to agricultural crops [1]. One such species is the Mediterranean pocket gopher (*Microtus Duodecimcostatus*), whose natural habitat includes the southeast of France and the northeast, central, and southern zones of the Iberian Peninsula. These pocket gophers are small underground rodents that weigh between 19 and 32 g; they are 9 to 11 cm long (head and body) with a tail of about 3 cm. These animals excavate corridors, which they use as shelter and breeding areas. The species is exclusively vegetarian and causes irreversible damage to agricultural crops.

Damage to agricultural crops has been reported by different authors. Apple trees are particularly susceptible to feeding damage during winter periods when voles feed on bark, vascular tissues and roots [2]. Populations of 1700 voles per acre (4250 voles/ha) in Washington State apple orchards decreased production by 35% [3]. In Europe, the common vole is a major vertebrate pest for plant production that can cause important economic losses during outbreaks [4]. In a survey carried out between 75 apple orchards in Germany [5], on 17% of the farms no damage occurred although vole species were present, 55% of the farmers reported a loss of up to 10% of apple trees/ha, 19% of the farmers lost up to a fourth and two farmers suffered from a total loss of trees. Damage occurred although the vole populations were controlled permanently.

Several techniques are currently used to combat pocket gophers: agricultural implements (burrow builders) pulled by a tractor to apply rodenticide bait underground; manual equipment to locally apply rodenticide bait underground; seasonal ploughing to destroy pocket gopher corridors; manual equipment to generate shock waves (by combustion of gas mixtures) in pocket gopher corridors; fumigation equipment; trapping equipment; and biological control. Burrow builders pulled by a tractor are used most successfully when there are large fields that have extensive pocket gopher populations at high densities due to its higher working capacity over other techniques [6].

For this reason, for extensive crops and fruit orchards, the most common techniques to combat pocket gophers are based on the underground application of rodenticide baits [6,7] by using modified mole ploughs [8]. In these techniques, the application depth and proper burial of the bait (without tilling) are the key factors for successful treatment [9]. A second work parameter, forward speed, is important because it is directly related to the time used to carry out the work. Importantly, valid solutions for a certain type of pocket gopher (genus *Microtus*) are not applicable to other types (genus *Thomomys, Talpa*, or *Arvicola*); therefore, specific solutions must be analysed for each type of pocket gopher.

Existing equipment for underground application of rodenticide baits were designed in USA [6] to fight voles of the genus *Thomomys* that are large rodents, between 15 and 30 cm. The equipment creates an underground tunnel of large diameter using a torpedo tube and produces a tilling of the land. Subsequently, this machine has been used in Europe for the fight against other smaller voles, in this case genus *Avicola* and *Talpa* [10], both larger than the genus *Microtus* (Mediterranean vole). The application of this machine in combating the Mediterranean vole has not been successful mainly due to the excessive size of the tunnel generated and the difficulty of producing a closure thereof.

Research carried out to develop agricultural implements has provided information on the force exerted by the ground over furrow opening systems depending on the type of soil [11], the working speed and depth [12], and the characteristics of implements such as mouldboard ploughs [13], mole ploughs [8], shallow ammonia injectors [14], spot ploughs [15], weed harrows [16], and seeders [17]. However, developing an agricultural implement to apply rodenticide bait against the Mediterranean pocket gopher requires specific research to optimise the performance of the machine. In this context, numerical analysis by the Finite Element Method (FEM) is a common technique used to design and develop vehicles as well as machine prototypes [18,19]. The technique is also used to design agricultural implements [20,21].

The FEM allows one to simulate the behaviour of a machine under working conditions. Once a machine is developed, experimental data are required to validate the design phase and to understand in detail the effect of the working conditions on the mechanical behaviour of the implement. To analyse the mechanical behaviour of a machine in the field, several types of sensors are used, including variable displacement transducers, potentiometers, and strain gauges. The use of strain gauges is widespread in the agricultural machinery sector [21,22] because such gauges are the most cost-effective and reliable solution to measure material stress in the field [23]. The gauges also supply information about deformations of the implement structure and can be calibrated as force transducers [15]. The numerical analysis allows one to locate the optimal areas for strain gauge placement and perform optimal comparative analyses of numerical and experimental results.

The main objective of this research was to design and test an agricultural implement for underground application of rodenticide bait in fruit orchards. To this end, a machine prototype was developed as a first step by using FEM and considering a range of loads generated on most commonly used furrow openers in agricultural implements. As a second step, the prototype was tested in the field to validate the design by analysing the effects of forward speed and application depth on the mechanical behaviour of the implement structure.

## 2. Experimental Section

### 2.1. Prototype Design

Numerical techniques have been employed to design the prototype. In this phase, a software based in the FEM has been used, concretely the software Abaqus 6.12 (DS Simulia—Providence, RI, USA). As a result, an agricultural implement prototype for the underground application of rodenticide bait was developed and fabricated.

The prototype, designed to apply rodenticide bait in fruit orchards, is composed of two parts: a fixed chassis and a mobile chassis. The fixed chassis is attached to the tractor linkage system. The mobile chassis is designed to revolve around one of the ends of the fixed chassis so that the machine is reduced in width and be driven on public roads. This machine design allows the operator to place the rodenticide bait dispenser near the tree line. This area is inhabited by the Mediterranean pocket gophers owing to its proximity to the drip lines.

The rodenticide bait is applied in a completely mechanical fashion. The implement consists of a furrow opener linked to a bait dispenser so that the product is deposited underground in the area near the tree line. Once the bait is deposited, a metal roller closes the furrow. The metal roller also acts as the drive element of the dispenser.

**Figure 1.**
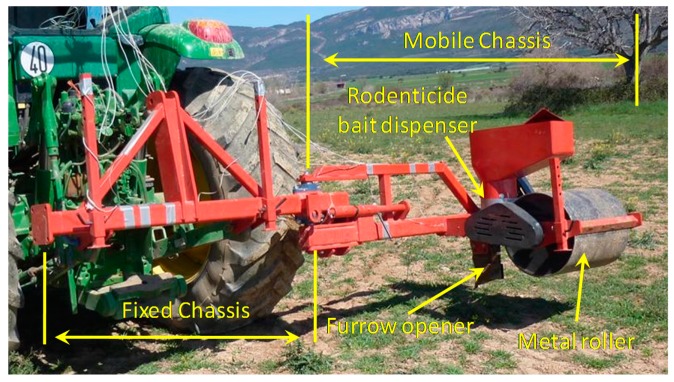
Components of the agricultural implement prototype: (1) fixed chassis; (2) mobile chassis; (3) furrow opener; (4) rodenticide bait dispenser; (5) metal roller.

The mobile chassis of the agricultural implement consists of the following components (Figure 1): furrow opener, rodenticide bait dispenser, and metal roller to close the furrow and to activate the rodenticide bait dispenser. The furrow opener consists of a rigid tine, 2 cm in width. A narrow tine was selected with the goal of create tunnels in line with the size of the Mediterranean vole. The implement was designed to work at a depth of 15 cm according to previous experiences carried out in Europe to fight against small voles by applying underground rodenticide bait [10]. The bait dispenser consists of a horizontal rotating disk with holes, placed on the bottom of a rodenticide bait reservoir. The disk receives the turning movement through a mechanical transmission driven by the metal roller. The rotating disk delivers the bait, located in the holes, to the tunnel generated by the furrow opener.

In the first phase of the numerical analysis, a series of documents [17,24–26] were consulted to establish the furrow opener geometry. The chassis geometry was set according to the experience of the research group and existing designs of implements with a certain similarity [8,14]. Once the geometry was completed, a finite element model of the agricultural implement was discretised.

**Figure 2.**
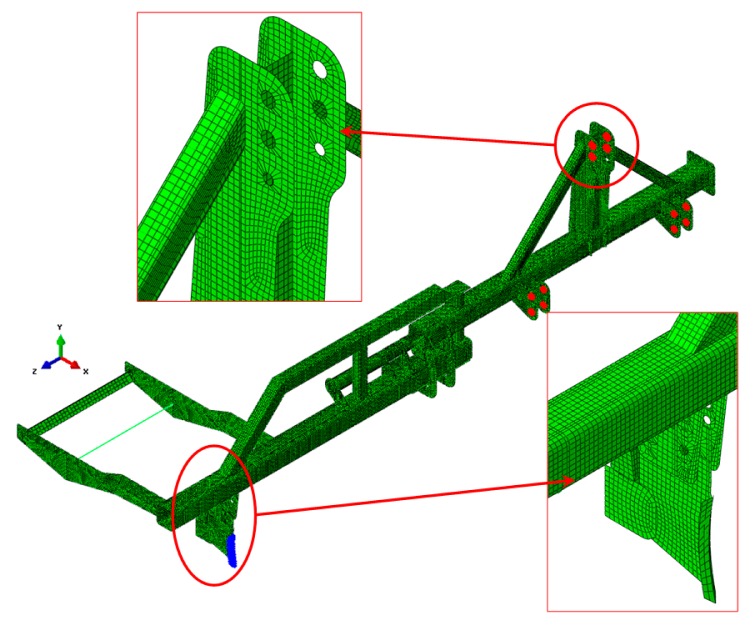
Finite element model of the prototype for underground application of rodenticide bait. Areas of load application (furrow opener, in blue) and boundary conditions (attachment to the tractor, in red).

The numerical model, shown in Figure 2, consists of 44,665 nodes and 43,399 elements. The elements used were of the shell type in all components except the metal roller, which was discretised by bar elements and mass elements. The main dimensions of the prototype are shown in Table 1.

The boundary conditions imposed in the numerical analysis prevented movement in the three areas at which the agricultural implement was attached to the tractor. These areas are shown in red in Figure 2.

Five load cases were analysed, which correspond to typical values of forces considering different types of furrow openers at depths around 0.15 m [27]. Loads were applied at the furrow opener (in blue in Figure 2). The five load cases considered were 445, 800, 2610, 4500, and 5000 N. The components of the prototype were designed and manufactured by using ST-52 steel (yield strength, 360 MPa). Information provided by the numerical analysis allowed us to identify the critical and oversized areas of the prototype in the design phase. Furthermore, the numerical results allowed us to determine the number of strain gauges and optimal areas for their placement to obtain an optimal comparative analysis of numerical and experimental results.

**Figure 3.**
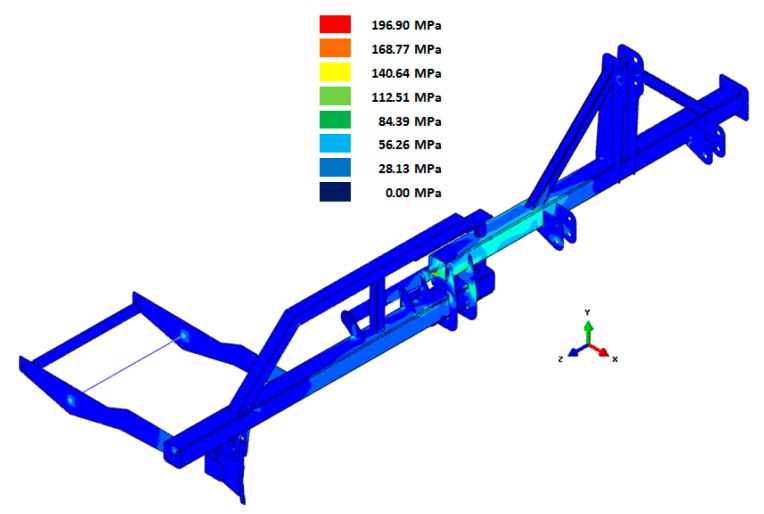
Results of Von Mises stress from the load case in which a force of 2610 N was applied to the furrow opener.

As an example, Figure 3 shows the Von Mises stresses obtained from the load case in which a force of 2160 N was applied to the furrow opener.

### 2.2. Experimental Measurements

The agricultural implement prototype was built once the numerical analysis by the FEM was completed. To record the strains in the prototype during the tests, a rosette (strain gauge 1) and three linear strain gauges (gauges 2–4) were installed in the prototype. The type and location of the strain gauge were determined from the results of the numerical analysis. Strain gauges supplied the experimental data required to validate the information obtained from the FEM simulation. Besides, the strain values let one analyze the effect of the forward speed and application depth on the structural behaviour of the implement.

The choice of sensor type depended on the stresses obtained in the numerical analysis. Specifically, linear strain gauges were used in areas in which the stress value in one direction was greater than the values in the other directions. In the case of the rosette, the numerical analysis showed that the in-plane stresses were similar for all directions.

**Figure 4.**
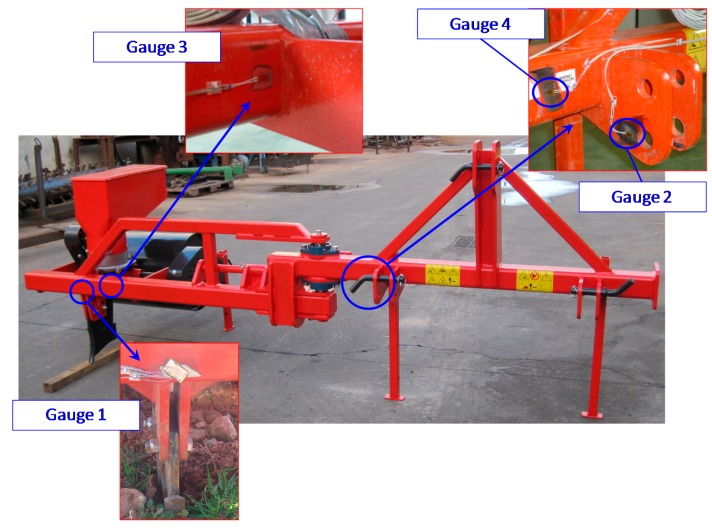
Strain gauge locations on the agricultural implement prototype during the experimental tests.

The four sensors were located in critical areas, in which the stress gradients were low. The locations of the strain gauges are shown in Figure 4. A fourth unidirectional strain gauge was installed to correct the effects of noise and temperature recorded by the strain gauges.

The experimental tests involved a combination of three variables: forward speed, application depth, and type of soil. Three *forward speeds* (4.5, 5.5, and 7.0 km/h), three a*pplication depths* (0.12, 0.15, and 0.17 m), and two types of soil (clayey-silty-loam and clayey-silty-sandy, Table 2) were considered. For each combination of variables, continuous measurements were taken considering a pathway of 400 m. Thus, 18 pathways of 400 m were tested in total, nine for each type of soil.

The strain gauge signals were recorded at a frequency of 5 Hz by a strain gauge measurement system (StrainBook/616, Measurement Computing, Norton, MA, USA). This equipment allows simultaneous measurements of eight channels. The measurement system was connected to a laptop computer equipped with data acquisition software (Waveview 7.15, Measurement Computing) that could save the strain values of each measurement channel in a different data file.

### 2.3. Statistical Analysis

The micro-strains obtained by the strain gauges were corroborated in their normality by the Kolmogorov-Smirnov test (*p* < 0.001) and their homoscedasticity with respect to the parameters *forward speed* and *application depth* by the Levene test (*p* < 0.001). Therefore, non-parametric methods were required for the analysis [28].

To carry out the study, the strain values obtained were treated as absolute values. A positive or negative value indicates that the gauge is being subjected to tension or compression, respectively.

The reliability of the strains obtained in the measurement channels was evaluated by a t-test. To ensure independence of observations, a bootstrap of 1000 samples without stratification was conducted [29].

Differences between the micro-strains obtained by the strain gauges depending on the field texture were statistically analysed by the Mann-Whitney U-test. The corresponding differences depending on the *forward speed* or *application depth* were analysed by the Kruskal-Wallis test. In the latter case, post-hoc analyses of differences between levels of each parameter were performed by the Mann-Whitney U-test.

## 3. Results and Discussion

### 3.1. Considerations of the FEM Design

The results of the experimental tests allowed to analyse the five initial hypotheses of load cases applied to the numerical analysis developed by the FEM. Table 3 shows the comparison between the numerical and experimental results obtained by gauges 1–4, corresponding to an application depth of 0.17 m considering the entire range of forward speeds and soil types. Data were acquired by using 8 measurement channels. In this sense, Channels 1–3 corresponded to the rosette (gauge 1). Channels 4, 6, and 7 corresponded to the three linear strain gauges (gauges 2–4, respectively). Channel 5 corresponded to a linear strain gauge, which was installed to correct the effects of noise and temperature recorded by the strain gauges.

The experimental tests allowed validation of the prototype design. The correlation between numerical and experimental results showed that the range of load hypotheses considered (445 to 5000 N) correctly represents the actual behaviour of the implement, considering that the experimental data corresponding to the application depth of 0.17 m were those at the worst working condition and the effect of velocity was insubstantial as shown below. Considering an application depth of 0.17 m (Table 3), the stresses and strains recorded in the field were similar to those simulated by numerical analysis at a load case of 5000 N which was the largest load case applied to the numerical model. This suggests the use of high load conditions in the design phase.

**Figure 5.**
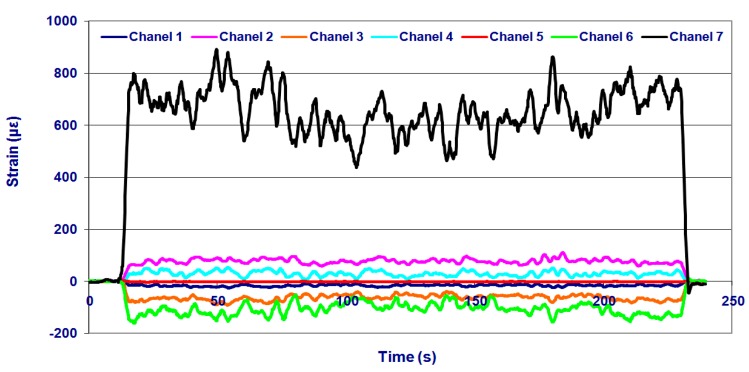
Strains (με) recorded in the experimental test in the field with loam texture (field 1), at a forward speed of 7 km/h and an application depth of 0.17 m.

Data recorded by the measurement gauges showed that the prototype structure behaved similarly in the two fields tested, with higher strain values for the loam texture (field 1). The strains recorded by Channel 7 were the highest among all channels in both fields. Channel 6 corresponded to the linear strain gauge placed in the main bar of the fixed chassis (gauge 4). These strains are caused by bending due to the force applied at the furrow opener. The highest strains were recorded in field 1. The strains recorded in this field, at a forward speed of 7 km/h and an application depth of 0.17 m, are shown in Figure 5. Of all possible configurations, this configuration yielded the maximum strains in all gauges. The Von Mises stresses calculated from the data registered by the strain gauges for this configuration are shown in Table 4. The minimum safety coefficient of all components was 1.9. These results, in addition to the numerical analysis developed, show that the prototype design is valid.

The results obtained in the experimental tests validated the design of the prototype which was able of creating narrow tunnels with the furrow opener of 2 cm width at different forward speeds and application depths. The methodology of simulating the performance of the implement by introducing static loads on the furrow opener with a FEM model was appropriate because the numerical results were well correlated to those obtained in the experimental tests.

### 3.2. Effect of Forward Speed and Depth

**Figure 6.**
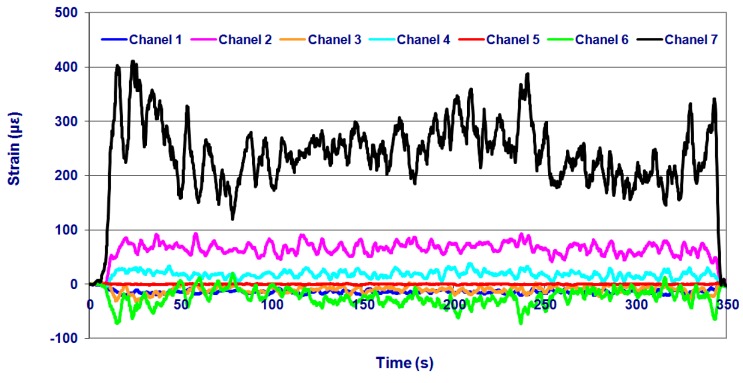
Strains (με) recorded in the experimental test in field 2 (sandy) at a *forward speed* of 4.5 km/h and an *application depth* of 0.15 m. Rosette: Channels 1–3; 3 Linear strain gauges: Channels 4, 6, and 7; Reference strain gauge: Channel 5.

The effects of *forward speed* and *application depth* on the mechanical performance of the implement chassis were analysed by considering the information obtained from the strain gauges. As an example, Figure 6 shows the micro-strains recorded by the strain gauges for a pathway of 400 m in field 2 (sandy) at a *forward speed* of 4.5 km/h and an *application depth* of 0.15 m.

The micro-strains measured by the reference gauge were not significantly different from zero (0.0109 ± 0.00607; Bias bootstrap = −0.33 × 10^−4^, *t* = 1.673, *p* = 0.075). Therefore the measurements obtained in the other gauges were considered as valid.

**Figure 7.**
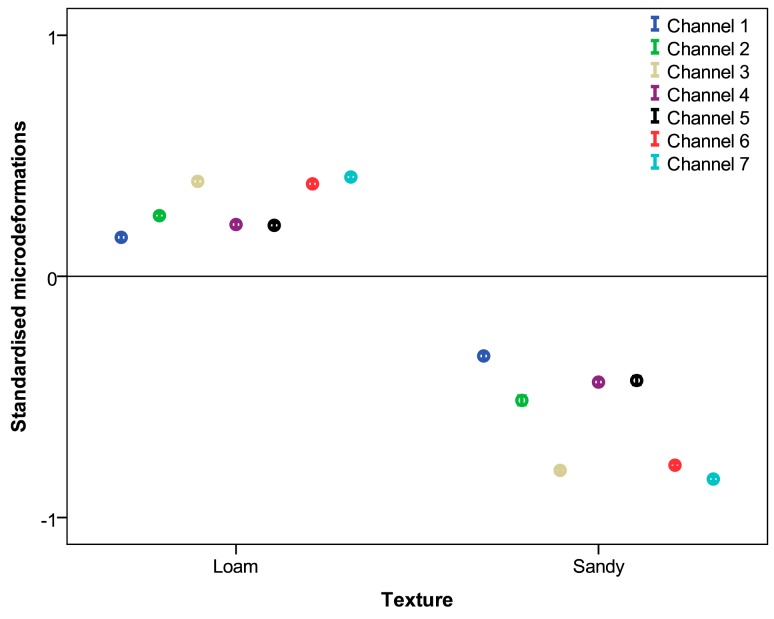
95% confidence intervals of all micro-strains standardised according to soil type.

Table 5 shows the means and standard deviations of the micro-strains recorded by the seven measurement channels in the two fields tested. The table shows that the strains obtained from the field with loam texture are significantly higher than the results recorded in the field with sandy texture. In addition, the strains obtained by the seven gauges vary widely. This variation is expected because the gauges are placed in different components of the chassis, which operate differently. The 95% confidence intervals of the micro-strains of each gauge, standardised according to the soil type, are shown in Figure 7.

Tables 6 and 7 show the average micro-strains measured by the gauges as a function of the forward speed and application depth, respectively. The analysis results show that micro-strains recorded at different forward speeds were significantly different in all gauges. Maximum values were registered at low velocities for Channels 1–5 and at the maximum velocity for Channels 6 and 7. However, although the differences were significant, the micro-strains did not show great variation depending on the *forward speed* from the viewpoint of designing the structure of the machine.

**Figure 8.**
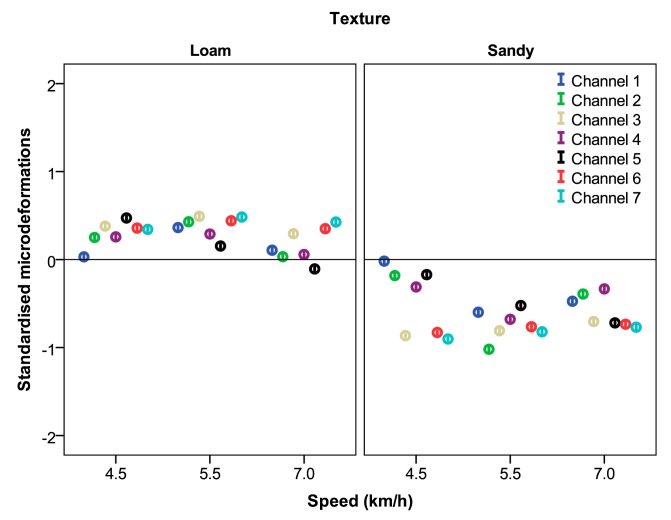
95% confidence intervals of all micro-strains standardised according to soil texture at different forward speeds.

**Figure 9.**
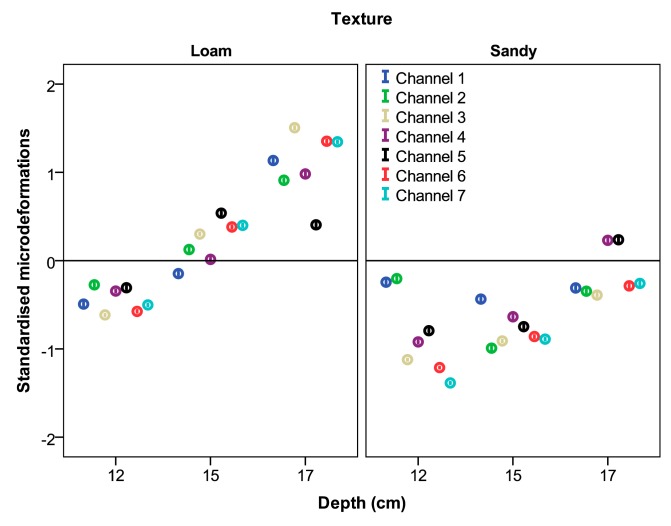
95% confidence intervals of all micro-strains standardised according to soil texture at different application depths.

Micro-strains obtained at different working depths were significantly different for all strain gauges. In addition, the differences increased as the *application depth* increased. In this case, the micro-strains showed a great variation depending on the *application depth* from the design perspective. Most gauges showed an increment in the recorded values bigger than 50% when the *application depth* changed from 0.12 to 0.17 m. The 95% confidence intervals of the micro-strains of each gauge standardised according the soil texture at different *forward speeds* and the *application depths* are shown in Figures 8 and 9, respectively.

The results showed that the *application depth* had a greater effect on the behaviour of the chassis than did the *forward speed*. In addition, the effects of the *application depth* were linear: as the *application depth* increased, the strains in the chassis also increased. In contrast, as the *forward speed* increased, the strains on the chassis remained roughly constant.

These findings are concordant with those found in other studies. Draught forces increase substantially for a tine with increased depth, both on open soil [30] and in grassland [31,32]. The draught force for rigid tines reportedly increases linearly with speed for sandy clay loam and is linear with a discontinuity for clay [33]. Others studies have not found an increase in forces for injector tines with increased speed [34].

## 4. Conclusions

A prototype device for underground application of rodenticide bait to control the Mediterranean pocket gopher in fruit orchards was designed and tested by using the FEM. Strain gauges were used in the field to validate the design phase and yield information on the structural performance of the implement at different speeds and application depths. Both forward speed and depth had a significant effect on the mechanical behaviour of the chassis. In particular, the application depth was the critical parameter that affected the strains on the structure of the implement. The effects of the application depth on the strains were linear such that strains increased with depth. In contrast, strains remained roughly constant regardless of the forward speed.

## Figures and Tables

**Table 1.** Dimensions of the prototype.

**Prototype Dimensions**	**Value** (**m**)
Fixed Chassis Width	1.750
Mobile Chassis Width	1.844
Distance between the bottom of the fixed chassis and the bottom of the mobile chassis	0.19
Distance between the bottom of the mobile chassis and the bottom of the furrow opener	0.38
Working distance (Distance between the centre of the tractor and the furrow opener)	2.110

**Table 2.** Properties of the soils corresponding to the trial plots.

**Field**	**Humidity (%)**	**Soil Type**	**Texture**
1	9.3	clayey-silty-loam	16.1% sand; 49.3% loam; 35.6% clay
2	8.1	clayey-silty-sandy	45.5% sand; 23.3% loam; 31.2% clay

**Table 3.** Stresses and strains registered by the gauges for numerical and experimental results corresponding to an application depth of 0.17 m, considering the entire range of forward speeds and soil types.

**Gauge**	**Load Cases Considered in the Numerical Analysis** (**FEM**)					**Experimental Results**

	**445 N**	**800 N**	**2610 N**	**4500 N**	**5000 N**	
1 (rosette)	2.14 MPa	2.98 MPa	9.49 MPa	16.98 MPa	17.81 MPa	17.61 MPa
2 (linear)	3.87 μξ	6.77 μξ	16.25 μξ	32.66 μξ	36.01 μξ	40.23 μξ
3 (linear)	−17.28 μξ	−22.25 μξ	−39.69 μξ	−79.88 μξ	−85.13 μξ	−88.76 μξ
4 (linear)	60.09 μξ	92.81 μξ	261.02 μξ	481.00 μξ	541.10 μξ	549.40 μξ

**Table 4.** The von Mises stresses and safety coefficients obtained from measurements registered by the strain gauges in the field with loam texture (field 1), at a forward speed of 7 km/h and an application depth of 0.17 m.

**Strain Gauge**	**Stress(MPa)**	**Yield Strength(MPa)**	**Safety Coefficient**
1	45.81	360.00	7.86
2	0.68	360.00	529.10
3	33.48	360.00	10.75
4	187.27	360.00	1.92

**Table 5.** Means and standard deviations of micro-strains (με) measured by gauges depending on the soil tested. Mean differences tested with the Mann-Whitney U-test. SS: Standardised Statistic.

**Measurement Channel**	**Soil**		**Probability**	

	**1: Loam** (***n*** = **25,148**)	**2: Sandy** (***n*** = **12,322**)	**SS**	***p***
1	13.937 ± 5.373	11.477 ± 3.628	−41.382	<0.001
2	67.595 ± 15.465	54.131 ± 18.140	−63.186	<0.001
3	41.054 ± 22.446	13.053 ± 10.258	−119.220	<0.001
4	31.757 ± 17.167	21.043 ± 11.806	−60.822	<0.001
5	1.758 ± 0.955	1.126 ± 0.895	−63.235	<0.001
6	72.241 ± 36.945	26.839 ± 20.938	−111.349	<0.001
7	479.399 ± 156.796	260.570 ± 102.492	−118.589	<0.001

**Table 6.** Average micro-strains (με) measured by gauges at different forward speeds. One-way ANOVA: forward speed (km/h). Mean differences tested with the Kruskal-Wallis test. Different letters indicate significant differences (*p* < 0.05) obtained through the Mann-Whitney U-test for a specific measurement channel considering different forward speeds.

**Measurement Channel**	**Forward Speed** (**km/h**)			**Probability**	

	**4.5** (**n** = **15,055**)	**5.5** (**n** = **12,482**)	**7.0** (**n** = **9,933**)	**Chi-Square**	***p***
1	13.200 ± 4.342b	13.373 ± 5.888c	12.711 ± 4.699a	53.771	<0.001
2	65.039 ± 15.040b	62.391 ± 22.796b	61.305 ± 12.622a	472.309	<0.001
3	31.042 ± 23.714a	33.403 ± 24.290c	31.107 ± 21.463b	78.746	<0.001
4	29.346 ± 19.420b	27.802 ± 15.635ab	27.089 ± 11.507a	14.539	0.001
5	1.803 ± 1.153c	1.484 ± 0.844b	1.249 ± 0.729a	1,489.202	<0.001
6	55.857 ± 38.568a	59.155 ± 40.071c	57.195 ± 37.898b	40.770	<0.001
7	394.909 ± 170.765a	417.428 ± 180.190b	413.871 ± 172.470b	143.294	<0.001

**Table 7.** Means and standard deviations of micro-strains (με) measured by gauges at different application depths. Mean differences tested with the Kruskal-Wallis test. Different letters indicate significant differences (*p* < 0.05) obtained through the Mann-Whitney U-test for a specific measurement channel considering different depths.

**Measurement Channel**	**Depth** (**cm**)			**Probability**	

	**12** (**n** = **12,468**)	**15** (**n** = **12,250**)	**17** (**n** = **12,482**)	**Chi-Square**	**p**
1	11.068 ± 3.723a	11.917 ± 4.265b	16.401 ± 5.163c	7,374.958	<0.001
2	58.751 ± 11.747a	58.932 ± 21.119b	71.826 ± 15.139c	5,013.109	<0.001
3	13.624 ± 8.225a	29.596 ± 15.610b	52.303 ± 23.900c	16,229.685	<0.001
4	19.501 ± 9.333a	24.967 ± 13.301b	40.234 ± 17.666c	10,368.368	<0.001
5	1.092 ± 0.684a	1.664 ± 1.055b	1.893 ± 0.985c	4,382.641	<0.001
6	26.852 ± 18.524a	56.291 ± 30.119b	88.756 ± 37.141c	15,540.010	<0.001
7	269.405 ± 94.144a	403.358 ± 133.349b	549.405 ± 160.432c	15,625.539	<0.001
